# Striatal activity and reduced white matter increase frontal activity in youths with family histories of alcohol and other substance-use disorders performing a go/no-go task

**DOI:** 10.1002/brb3.352

**Published:** 2015-05-28

**Authors:** Ashley Acheson, Malle A Tagamets, Anderson Winkler, Laura M Rowland, Charles W Mathias, Susan N Wright, L Elliot Hong, Peter Kochunov, Donald M Dougherty

**Affiliations:** 1 Department of Psychiatry, University of Texas Health Science Center at San AntonioSan Antonio, Texas; 2 Research Imaging Institute, University of Texas Health Science Center at San AntonioSan Antonio, Texas; 3 Department of Psychiatry, Maryland Psychiatric Research Center, University of Maryland School of MedicineBaltimore, Maryland; 4 Oxford Centre for Functional MRI of the Brain, University of OxfordOxford, UK; 5 Department of Psychiatry, Yale University School of MedicineNew Haven, Connecticut; 6 Russell H. Morgan Department of Radiology and Radiological Science, Johns Hopkins UniversityBaltimore, Maryland

**Keywords:** Family history, functional magnetic resonance imaging, go/no-go task, risk, substance use, white matter integrity

## Abstract

**Introduction:**

Youths with a family history of alcohol and other drug use disorders (FH+) are at greater risk of developing substance-use disorders relative to those with no such family histories (FH−). We previously reported that FH+ youths have elevated activity in the supplementary motor area (SMA) and dorsal striatum while performing go/no-go tasks and have reduced frontal white matter integrity. A better understanding of relationships between these variables would provide insight into how frontostriatal circuitry is altered in FH+ youths, which may be an important contributor to their elevated risk.

**Methods:**

In this study, we used structural equation modeling (SEM) to test interactions between activity in the SMA and dorsal striatum in 72 FH+ and 32 FH− youths during go/no-go task performance and to determine whether increased activity in these regions in FH+ youths can be at least partially explained by reduced frontal white matter integrity, as indexed by anterior corona radiata fractional anisotropy and N-acetylaspartate.

**Results:**

Increased dorsal striatum activity explained most (∽75%) of the elevated SMA activity in FH+ youths, and the combined contributions of increased dorsal striatal activity, and decreased white matter integrity fully explained the elevated SMA activity.

**Conclusions:**

These results suggest the elevated frontal cortical activity in FH+ youths is driven both by their increased striatal activity via downstream projections and reduced white matter integrity in frontal cortical projections, the latter likely increasing frontal cortical activity due to increased energy demands required for action potential propagation. As part of our ongoing longitudinal studies we will examine how these frontostriatal alterations relate to risk for developing substance-use disorders.

## Introduction

**Figure 1:**
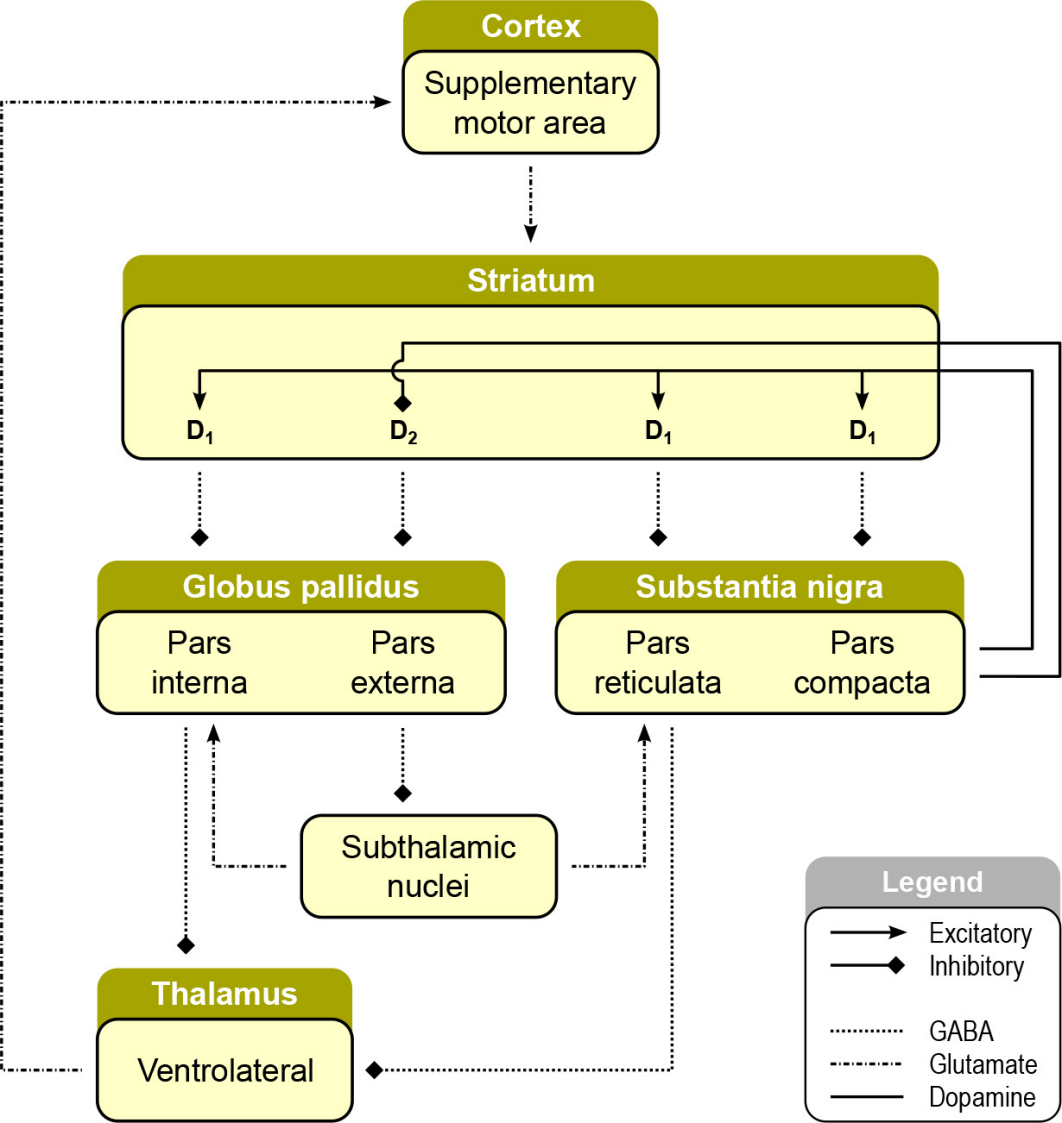
Schematic depiction of the frontostriatal motor loop. The supplementary motor area sends excitatory glutamatergic input to the striatum, which sends inhibitory GABAergic projections to globus pallidus and substantia nigra. Both these regions have inhibitory GABAergic projections to the ventrolateral thalamus, with the substantia nigra sending dopaminergic input back to the striatum that can have either excitatory (dopamine receptor D1) or inhibitory (dopamine rector D2) influences on striatal firing. The ventrolateral thalamus projects back to the supplementary motor area, completing the loop.

Adolescents and young adults with family histories of alcohol and other drug use disorders (FH+) are at greatly increased risk for substance-use disorders relative to those with no family histories of substance-use disorders (FH−; Sher et al. 2004; Sher and Trull 1994; Tarter et al. 2003). This risk may be at least partially due to frontostriatal circuitry dysregulations as FH+ youths and young adults have: (1) reduced frontostriatal white matter development (Herting et al. 2010; Acheson et al. 2014b,c); (2) altered and often increased activity in frontostriatal and other forebrain regions (e.g., Schweinsburg et al. 2004; Spadoni et al. 2008; Acheson et al. 2009; Cservenka et al. 2012, 2014); and (3) impaired cognitive processes regulated by frontostriatal circuitry such as impulse control (e.g., Corral et al. 2003; Stevens et al. 2003; Lovallo et al. 2006; Acheson et al. 2011a,b; Dougherty et al. 2014). One commonly used functional imaging paradigm that activates frontostriatal circuitry is the go/no-go task, which requires selective responding to appropriate stimuli (“go” trials) and inhibiting responding to inappropriate stimuli (“no-go” trials). Recently, we tested a large cohort of FH+ and FH− youths performing a go/no-go task and found FH+ youths had robustly elevated frontostriatal activity during task performance with activation peaks in the supplementary motor area (SMA) of the medial superior frontal gyrus and dorsal striatum (Acheson et al. 2014a). Both the SMA and dorsal striatum are part of the frontostriatal motor loop (Chudasama and Robbins 2006; Tekin and Cummings 2002; see Fig.1) and are key regions implicated in performing go/no-go tasks and similar measures involving selective attention and response inhibition (see Eagle et al. 2008 for reviews).However, it is not clear what is driving the increased activity in these regions in the FH+ youths. In this study, we used structural equation modeling (SEM) analyses on to test: (1) how elevated activity in the SMA and dorsal striatum influence each other; and (2) how reduced frontal white matter integrity contributes to the increased SMA and dorsal striatum activity.

In the frontostriatal motor loop (Fig.1), the SMA sends excitatory glutamatergic input to the dorsal striatum, which sends inhibitory GABAergic projections to globus pallidus and substantia nigra. Both of these regions have inhibitory GABAergic projections to the ventrolateral thalamus, with the substantia nigra sending dopaminergic input back to the striatum that can have either excitatory or inhibitory influences on striatal firing. Finally the thalamus projects back to the SMA, closing the loop. Thus, the increased SMA activity we observed may be driving the increased dorsal striatal activity via its direct glutamatergic inputs to this region. Alternatively, the increased dorsal striatum activity may be leading to increased SMA activity by reducing inhibition of the ventrolateral thalamus, thus facilitating glutamate release to the SMA.

Additionally, the increased frontostriatal activations we observed in FH+ youths during go/no-go task performance appear to be partially driven by their reduced frontostriatal white matter integrity. Increased forebrain activations have previously been associated with reduced white matter integrity and are thought to reflect decreased neural efficiency (Burzynska et al. 2013; Zhu et al. 2015). Specifically, reduced white matter integrity would result in more ions leaking through axonal membranes and increase the metabolic burden for action potential propagation on neurons due to impaired saltatory conduction (Bartzokis et al. 2003, 2004, 2010; Bartzokis 2004). Therefore, because of reduced white matter integrity, the FH+ youths may require more processing effort and energy utilization in neurons for communication between distal brain regions, resulting in increased functional activations.

In this study we used SEM analyses on previously published go/no-go activation data from our cohort of FH+ and FH− youths (Acheson et al. 2014a) to examine mechanisms underlying the increased SMA and dorsal striatum activity in FH+ youths during go/no-go task performance. We tested to what extent FH+ youths increased activity in each region could be explained by increased activity in the other region. We also tested to what extent the decreased frontal white matter integrity in these FH+ youths that we previously reported (Acheson et al. 2014b,c) contributed to the increased activity in these regions. We looked at frontal white matter integrity in the anterior corona radiata, which includes projections from the SMA to the dorsal striatum. White matter integrity was indexed with fractional anisotropy, which estimates myelin levels, and N-acetylaspartate, which is a neurochemical important for the development and maintenance of cerebral myelination and axonal health (Moffett et al. 2007).

## Methods

### Participants

Seventy two FH+ and 32 FH− youths (10 to 14 years old, average age = 12.9 ± 1.0) were recruited from a cohort of 386 volunteers in an ongoing longitudinal study on adolescent development and substance-use involvement (Acheson et al. 2014a,b,c; Ryan et al. In Press[Bibr b38]). Eleven participants who were included in our white matter integrity papers (Acheson et al. 2014b,c) were excluded because they did not have usable fMRI data. Exclusion criteria included: regular substance use (defined as substance use at least once per month for 6 consecutive months; Clark et al. 2005), positive urine test at time of screening, low IQ (<70), or physical/developmental disabilities that would interfere with the ability to understand or complete study requirements. Oppositional Defiant Disorder, Conduct Disorder, ADHD, Dysthymia or Anxiety Disorders were not exclusionary for the FH+ group because these disorders are commonly comorbid with substance-use involvement (Iacono et al. 2008). The Institutional Review Board of The University of Texas Health Science Center at San Antonio approved the study procedures. Privacy was further protected by a Certificate of Confidentiality from the Department of Health and Human Services.

### Family history of substance-use disorders

Family history classification for the youth cohort was established using the Family History Assessment Module (Rice et al. 1995) based on parent report using DSM-IV-TR diagnostic criteria (APA, 2000). All FH+ participants had at least a biological father with a past or present substance-use disorder. Most FH+ youths (79%) had a father with an alcohol use disorder history, and 58% had a father with history of alcohol and other drug use disorders. Some FH+ youths (29%) also had a biological mother with substance-use disorder histories. Eighteen percent had a mother with an alcohol use disorder history and 11% had a mother with history of alcohol and other drug use disorders. The most common other drug use disorders among parents were cannabis and stimulant use disorders.

### Collection and processing of magnetic resonance imaging and spectroscopy data

Imaging was performed at the Research Imaging Institute, University of Texas Health Science Center at San Antonio. All imaging data were collected using a Siemens Tim Trio 3T MR system (Erlangen, Germany) equipped with a 12 channel head coil. This study combined functional magnetic resonance imaging (fMRI) collected during go/no-go task performance with two measurements of frontal white matter integrity derived from diffusion tensor imaging (DTI) and proton magnetic resonance spectroscopy (1H-MRS) (Wijtenburg et al. 2012). The two measures of white matter integrity used, fractional anisotropy of water diffusion and concentrations of the N-acetylaspartate from the anterior corona radiata, were included because they were shown to be reduced in FH+ subjects (Acheson et al. 2014b,c).

### Go/no-go fMRI task

The go/no-go task is a standard fMRI task for probing selective attention and inhibition networks, and we have previous reported on functional activations induced by this task version in this study cohort (Acheson et al. 2014a). This task consisted of 12 alternating 28 sec blocks of go trials only (Go Only blocks) with blocks of 50% go trials and 50% no-go trials (Go/No-Go blocks). Each block was preceded by a 2-sec instruction. In the go condition, subjects were presented with a series of random letters and are instructed to respond for all letters. In the no-go condition, subjects are shown random letters 50% of the time and the letter “D” the remaining trials and are instructed to respond for each letter except for the letter “D”. Within all blocks (Go Only and Go/No-Go), stimulus presentation was 0.5 sec with an interstimulus interval of 1.5 sec. The fMRI task was performed using a gradient-echo, echo-planar sequence, acquiring 43 continuous slices parallel to the anterior commissure-posterior commissure (AC-PC) plane (repetition time/echo time [TR/TE] = 3000/30 msec, 1.72 × 1.72 × 3.0 mm, and field of view [FOV] = 220 mm). For anatomical reference, a 3-D high-resolution T1-weighted series was acquired (TR/TE = 2000/2.83 msec, flip angle = 13°, 0.8 × 0.8 × 0.8 mm, FOV = 256 mm) using an optimized protocol described previously (Kochunov et al. 2006).

### fMRI data preprocessing and data extraction

Preprocessing was performed in SPM8 (Wellcome Department of Imaging Neuroscience, London) and consisted of slice timing correction, motion correction, spatial normalization, and spatial smoothing with an 8 mm FHWM Gaussian filter. A first-level analysis was performed to generate Go/No-Go task versus baseline contrast maps for each subject. Previously, we found robust between FH group differences in both Go and Go-No-Go activations vs baseline (Acheson et al. 2014a). The goal of the present analysis was to examine the connectivity of a network and to examine whether white matter integrity differences contribute these activation differences. These were entered into a one-sample *t*-test that included both groups of subjects. Data for the structural equation modeling were derived from these results by extracting the first eigenvariate of a 12-millimeter-diameter sphere centered on each region of interest (ROI) at the coordinates of the peak activation reported in Acheson et al. (2014a).

### Frontal white matter integrity measurements

Fractional anisotropy values and N-acetylaspartate concentrations measurements of white matter integrity were performed for the anterior corona radiate as described elsewhere (Wijtenburg et al. 2012; Acheson et al. 2014c). In short, fractional anisotropy was measured using a single-shot, echo-planar, single-refocusing spin-echo sequence to acquire diffusion-weighted data with a spatial resolution of 1.7 × 1.7 × 3.0 mm. The sequence parameters were: TE/TR = 83/7000 msec, FOV = 200 mm, two diffusion weighing values *b* = 0 and 700 sec/mm^2^ and five *b* = 0 (nondiffusion weighted) images, 64 isotropically distributed diffusion-weighted directions, and axial slice orientation with 50 slices and no gaps. The number of directions, *b* = 0 images, and the magnitude of the *b* values were calculated using an optimization technique that maximizes the contrast to noise ratio based on the average diffusivity of the cerebral white matter and the *T*_2_ relaxation times (Jones et al. 1999). Fractional anisotropy images were created by fitting the diffusion tensor to the raw diffusion data (Pierpaoli et al. 1996; Jenkinson et al. 2012). The DTI data were processed using a tract-based spatial statistics (TBSS) analytic method, distributed as a part of the FMRIB Software Library (FSL) package (Smith et al. 2006, 2007).

Concentrations of N-acetylaspartate were measured using a single voxel PRESS localization sequence with the following parameters as described previously: TR/TE = 1500/135-msec, voxel of interest (VOI) = 15 × 15 × 15 mm, NEX = 256, 1024 complex points, 1.2 k-Hz spectral width, and total scan time = 12 min (Acheson et al. 2014b). A water reference (NEX = 4) was collected and utilized for phasing and eddy current correction. Spectroscopic voxels were placed bilaterally in the forceps minor area of each hemisphere to prevent contamination of partial volume, cerebrospinal fluidor gray matter. MRS data in all subjects were collected by the same technician who ensured the correct placement of the spectroscopic voxel in pure frontal white matter. All data were phased and apodized to improve the signal-to-noise ratio using an in-house IDL program (Exelis Visual Information Solutions, Inc, Boulder, CO) (Wijtenburg and Knight-Scott 2011).

### Structural equation modeling methods

Structural equation modeling (SEM) was used to examine relationships between increased activity in the left SMA (BA 6/8; −10, 16, 64) and right dorsal striatum (18, 20, −6) in FH+ youths during go/no-go task performance and to test the hypothesis that increased activations are at least partially explained by reduced frontal white matter integrity. The left SMA was selected because FH+ youths had significantly elevated activity here in both the Go and No-Go minus baseline subtractions, and the right dorsal striatum was selected because FH+ youths had significantly elevated activity here in the Go minus baseline subtraction and just subthreshold elevated activity here in the No-Go minus baseline subtraction (Acheson et al. 2014a). Models were constructed from these regions and one structural (fractional anisotropy) and one chemical (N-acetylaspartate) measure of white matter integrity from the anterior corona radiata (Acheson et al. 2014b,c). The anterior corona radiata was chosen because it carries connections between the frontal cortex and striatal areas. The fractional anisotropy and N-acetylaspartate data were normalized to same units of variance as the fMRI data to avoid biasing the SEM analyses.

### Modeling procedures

**Figure 2:**
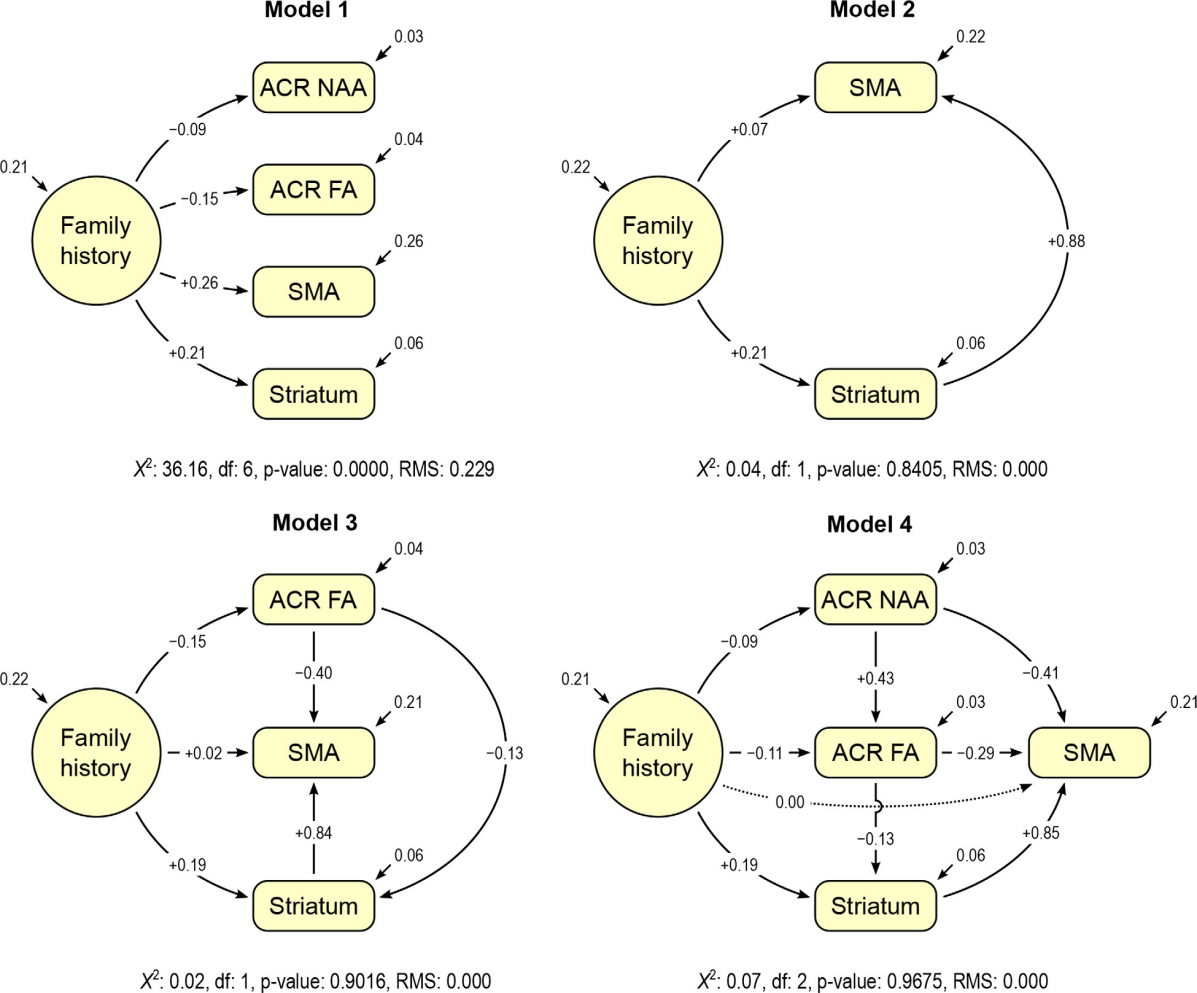
Model 1 tested direct effects of family history on supplementary motor area (SMA) and dorsal striatum activity and white matter integrity (anterior corona radiata (ACR) fractional anisotropy (FA) and N-acetylaspartate (NAA). Family history was significantly associated with functional activations and white matter integrity measures, but the overall model was not statistically significant (*P* = 0.00%, RMSEA = 0.229 [0.160; 0.303], AIC = −664; CFI = 0.706). Model 2 tested simple modulation effects of dorsal striatal activity on SMA activity. This model showed a significantly improved fit compared to Model 1 (*P* = 84%, RMSEA = 0.000 [0.0; 0.158]; AIC = −251; CFI = 1.00) and reduced the strength family history on SMA activity, from 0.26 to 0.07 (73% reduction). Model 3 tested effects of fractional anisotropy on modulating activity in the SMA and dorsal striatum. This model showed an improved fit (*P* = 90%, RMSEA = 0.000 [0.0; 0.119], AIC = −446; CFI = 1.00) and further reduced the influence of family history compared to Models 1 and 2. In particular, adding ACR FA to the model reduced the effect of family history on SMA activation by 72% when compared to Model 2. Model 4, includes NAA influences on ACR FA and SMA activity. The combined influences of striatal activation, ACR FA, and NAA account for 100% of the relationship between family history and SMA activity. This model also had the best fit (*P* = 97%, RMSEA = 0.000 [0.0; 0.0], AIC = −451; CFI = 1.00). Abbreviations: RMSEA, Root Mean Square Error of Approximation; numbers in square brackets following the RMSEA are the 90% confidence intervals; AIC, Akaike's Information Criterion; CFI, Comparative Fit Index.

The modeling proceeded in four stages (Fig.2). First, a model was created for estimating the dependence of all four variables on group membership. Next, we examined a model that included only SMA and dorsal striatum activations in order to determine how much variance is accounted for by functional interactions between these regions. We then examined how much additional variance in the SMA and dorsal striatum activations was explained by anterior corona radiata fractional anisotropy. Finally, anterior corona radiata N-acetylaspartate was added. In Fig.2, the direction of the arrows indicates direction of influence. The direction of influence from the fractional anisotropy and N-acetylaspartate data was always to the SMA and dorsal striatum activations, indicating that it is expected that white matter integrity influences activations and not vice versa. Likewise, we chose the connections from N-acetylaspartate to fractional anisotropy based on the prior research that suggests that N-acetylaspartate plays an important role in the development and maintenance of cerebral myelination and axonal health (Moffett et al. 2007). For connections between the SMA and dorsal striatum, directionality was tested in both directions, i.e., from dorsal striatum for SMA and from SMA to dorsal striatum.

Group-level SEM analyses were performed using LISREL 9.1 (Skokie, IL). In order to assure robustness, the models were re-estimated from different initial values. Model fit was evaluated by several criteria in order to evaluate robustness. The most commonly reported fit indices are the Root Mean Squared Error (RMSE; McDonald, 1989) and a probability measure (*P*) based on a maximum likelihood ratio based chi-square test. An RMSE below 0.05 indicates a good fit and below 0.01 a very good fit. A higher *P*-value indicates a better fit between the model and the data, with 1.0 indicating a perfect fit. The Akaike Information Criterion (AIC; Akaike, 1987) was also computed for each model. The AIC considers the complexity of the model and penalizes overfitting. The formula used to compute the AIC is −2ln(L) + 2 m, where L is the likelihood of a good fit and m is the number of free parameters. More negative AICs indicate a better fit with less complexity. Finally, the Comparative Fit Index (CFI; Bentler 1990) is based on the chi-square difference between the null model and the current model while taking the degrees of freedom into account. The CFI can range from 0 to 1, and a CFI greater than 0.95 is generally taken to indicate a good fit.

## Results

### Participant characteristics

Demographic data are summarized in Table 1 and have been reported previously (Acheson et al. 2014a). The FH+ and FH− groups did not differ in age, race, or ethnicity or drug use. FH+ participants had lower IQ, socioeconomic status. Some FH+ participants had externalizing and internalizing disorders.

**Table 1:** Demographics

	FH− (*n* = 32)	FH+ (*n* = 72)
	Mean (SD)	Mean (SD)
Age	12.9 (1.1)	12.9 (1.0)
Wechsler Abbreviated Scale of Intelligence	102.0 (11.7)	96.5 (12.6)*
Four Factor Index of Socioeconomic Status	42.9 (10.0)	34.8 (12.9)*

	Number (%)	Number (%)
Gender		
Male	18 (56)	35 (49)
Female	14 (44)	37 (51)
Race		
African-American	1 (3)	10 (14)
Caucasian	31 (97)	60 (83)
Other	0 (0)	2 (3)
Ethnicity		
Hispanic/Latino	28 (88)	56 (78)
Non-Hispanic/Latino	4 (12)	16 (22)
Externalizing Disorders		
Attention Deficit Hyperactivity Disorder	0 (0)	14 (20)
Conduct Disorder	0 (0)	3 (4)
Oppositional Defiant Disorder	0 (0)	7 (10)
Internalizing Disorders		
Generalized Anxiety Disorder	0 (0)	6 (8)
Separation Anxiety Disorder	0 (0)	1 (1)
Specific Phobia	0 (0)	4 (6)
Lifetime alcohol & drug use (ever used)		
Alcohol	1 (3)	5 (7)
Marijuana	0 (0)	3 (4)
Tobacco	1 (3)	1 (3)
Other	0 (0)	0 (0)

* *P *<* *0.05.

FH+, family history of substance use disorders (SUDs); FH−, no family history of SUDs; WASI, Wechsler Abbreviated Scale of Intelligence; (Psychological Corporation, 1999); FFIS, Four Factor Index of Socioeconomic Status; (Hollingshead 1975).

### SEM modeling

SEM modeling was performed to investigate relative contributions to the FH differences in SMA and caudate/putamen activations on Go/No-go task performance from the following four factors: functional activations in the SMA and dorsal striatum, and two anterior corona radiata white matter integrity measurements: fractional anisotropy and N-acetylaspartate. SEM fit quality for four models was tested (Fig.1). Model 1 tested direct effect of family history on functional activations and white matter integrity. This simple model demonstrated significant associations between family history, functional activations and white matter integrity measures (Fig.1). These findings are consistent with prior results we reported (Acheson et al. 2014a,b,[Bibr b7][Bibr b6]). However, the overall model was not statistically significant (*P* = 0.00%, RMSEA = 0.229 [0.160; 0.303], AIC = −664; CFI = 0.706). Here and in the following, numbers in square brackets indicate the 90% confidence interval for the RMSEA.

Model 2 considered the simple modulation effect of dorsal striatal activity on SMA activity. This model showed a significantly improved fit compared to Model 1 (*P* = 84%, RMSEA = 0.000 [0.0; 0.158]; AIC = −251, CFI = 1.00). This model greatly reduced the strength of the family history influence on SMA activations, from 0.26 to 0.07, a 73% reduction (Fig.1). This suggested that dorsal striatal activation plays a significant role in mediating the observed group differences in SMA activation.

Next, Model 3 tested effects of anterior corona radiata fractional anisotropy on modulating activations in the SMA and dorsal striatum. This model showed an improved fit (*P* = 90%, RMSEA = 0.000 [0.0; 0.119], AIC = −446, CFI = 1.00) and further reduced the influence of family history compared to Models 1 and 2. In particular, adding fractional anisotropy to the model reduced the effect of family history on SMA activation by 72% when compared to Model 2.

Finally, Model 4 was constructed based on the physiological role that N-acetylaspartate plays in the maintenance of white matter integrity by modulating fractional anisotropy values (Fig.1D). Two pathways, N-acetylaspartate to striatum, and family history to SMA, were pruned from the final estimation, due to lack of significance. This model showed the highest fit quality, with a 97% probability of fit. This model also had the lowest AlC score, at −693. REMSA is 0.00 [0.0; 0.0], and CFI = 1.00. This addition also removes the remaining effect of family history on SMA activity, which SEM computed to have a value of 0 in this model, thus fully explaining group differences in SMA activation.

Alternate models were also tested. Adding the fractional anisotropy pathway to family history effects on SMA and dorsal striatum activity in Model 1 reduced the influence of family history on SMA activity by about 31%, but the fit was very poor, with probability *P* = 0.0002. A similar result was found when N-acetylaspartate pathways to SMA and dorsal striatum activity were added to Model 1: family history influence to SMA was reduced by about 24%, and probability of fit was 0.0001. Thus, although both fractional anisotropy and N-acetylaspartate partially mediated the relationship between family history and SMA activity without including dorsal striatal activity in the models, the poor fit makes these models unacceptable.

Reversing the direction of the pathway between the dorsal striatum and SMA in Model 4 also did not yield as good a fit as in the original model. The probability of fit was about 32%. Furthermore, the influence of family history on striatal activation was only reduced by about 28% in this manipulation. A similar result was found in reversing the direction of the striatal-SMA pathway in Model 2. Thus while striatal activation is a good candidate for mediating group differences in SMA, SMA is not a good mediator for striatal activation.

Collectively the SEM analyses suggest that the combination of dorsal striatal activity as well as anterior corona radiata fractional anisotropy and N-acetylaspartate (markers of white matter integrity) fully mediates the relationship between family history and activation in the SMA. In these models, the greatest influence on the relationship between family history and SMA came from the striatal activation, which explains about 75% of the difference between groups in SMA activation. Both fractional anisotropy and N-acetylaspartate were also found to partially mediate the influence of family history on SMA activation, but the combined effects of striatal activation, and N-acetylaspartate were required to fully explain group differences.

The same four models were re-estimated after eliminating the FH+ participants with psychiatric disorders (*n* = 28). Overall, the results were very similar to the models that included data from all participants. Dorsal striatum still had the greatest effect in explaining between-group differences in SMA and adding the measures of white matter integrity further explained the group differences. Additionally, all influence strengths were virtually identical except family history had almost double the influence on SMA activity and Model 4 only achieved partial mediation of family history influence on SMA activation (a reduction of about 55%).

## Discussion

In this study, we used structural equation modeling (SEM) to test interactions between frontal-striatal regions with elevated activity in FH+ youths during go/no-go task performance that we previously reported (Acheson et al. 2014a) and to determine whether increased activity in these regions can be at least partially explained by reduced frontalstriatal white matter integrity findings we previously reported in this same cohort (Acheson et al. 2014b,c). In these models, increased dorsal striatal activity explained most (∽75%) of the elevated SMA activity in FH+ youths, whereas increased SMA activity did not significantly explain their increased striatal activity. Both reduced anterior corona radiata fractional anisotropy and N-acetylaspartate were also found to partially mediate the increased SMA activity in FH+ youths, and the combined contributions of increased striatal activation, and decreased fractional anisotropy and N-acetylaspartate fully explained the elevated SMA activations. These results suggest the elevated SMA activity is driven both by increased striatal activity via downstream projections in the frontostriatal motor loop and by reduced white matter integrity in frontal cortical projections, the latter likely increasing SMA activity due to increased energy demands required for action potential propagation.

The present findings are novel and have important implications about frontostriatal functioning in FH+ youths. Our results suggest that the increased dorsal striatum activity in FH+ youths is not driven by the SMA. In contrast, the high meditation of SMA activity by striatal activity suggests striatal activity may be increasing SMA activity by blocking inhibition of the ventrolateral thalamus and thus promoting glutamate release to the SMA. It is not clear what drives the increased striatal activity in the FH+ youths, but dopaminergic inputs to this region are a plausible candidate. Elevated striatal activity is thought to contribute to elevated reward-seeking and risk-taking behavior, including increased propensity toward problem substance use during normal adolescent development (Ernst and Fudge 2009; Somerville and Casey 2010; Blakemore and Robbins 2012). Our results suggest striatal activity is exaggerated in FH+ youths relative to their FH− peers, which may potentially relate to the enhanced risk for problem substance use in this population.

In addition to increased dorsal striatal activity driving increased SMA activity in FH+ youths, our results indicate that reduced frontal white matter integrity also contributed to the increased SMA activity. Specifically fractional anisotropy, an indirect measure of white matter levels, and N-acetylaspartate, a neurochemical indicator of white matter health and integrity, explained the remaining increased SMA activity in FH+ youths that was not accounted for by increased striatal activity. Reduced forebrain white matter integrity is associated with increased forebrain activations and is thought to reflect decreased neural efficiencies (Burzynska et al. 2013; Zhu et al. 2015). Thus, because of decreased white matter integrity, SMA activity in FH+ youths may be increased because SMA neurons require increased energy utilization for action potential propagation. Potentially, elevated striatal activity and poor white frontostriatal matter integrity may cause FH+ individuals to have increased activity in other frontal cortical regions that are part of frontostriatal loops, such as anterior cingulate cortex during simulated gambling task performance (Acheson et al. 2009), and orbital and medial prefrontal cortex during go/no task performance cortex in FH+ individuals with problem alcohol use (Heitzeg et al. 2010).

There are several caveats to this study. Our results provide evidence for altered frontostriatal functioning in FH+ youths that is partially mediated by reduced frontal white matter integrity. Although the directionality of relationships we observe are theoretically plausible, they would need to be confirmed with experimental manipulation. While our results indicate the frontostriatal motor loop is affected during go/no-go task performance, it is presently unclear to what extent this generalizes to other frontostriatal loops. Additionally, our cohort included FH+ individuals with externalizing and internalizing psychiatric disorders because these conditions are more common in FH+ youths, dimensionally associated with FH+ phenotypes, and frequently comorbid with drug and alcohol abuse (34). However, we did observe a more robust relationship between SMA activity and family history when individuals with psychiatric disorders were excluded and this was not fully mediated by increased dorsal striatal activity and reduced white matter integrity, suggesting that other factors may also be contributing. While a sample size of 104 is considered relatively small for a SEM study, most SEM studies in imaging have far fewer subjects. A recent analysis suggests that increasing group size causes accuracy to asymptotically approach a limit in accuracy, with group sizes between 50 and 100 essentially undistinguishable from much larger sample sizes (Boucard et al. 2007). It was also shown that even the smallest sample sizes (about 30) generally preserve relative path coefficients even if the values are not exact. Finally, it is presently unclear how increased forebrain activations may relate to risk for developing substance-use disorders although it may be a marker for impaired or delayed forebrain development. Longitudinal studies are necessary to address these issues.

## Conclusions

In summary, we observed that increased frontal cortical activity we previously reported in FH+ youths performing a go/no-go task (Acheson et al. 2014a) appears to be driven both by increased striatal activity and reduced frontostriatal white matter integrity. We are tracking this cohort longitudinally as part of a study on adolescent brain and impulse control development and progression of substance-use disorders. As part of this study, we will evaluate how altered frontostriatal functioning contributes to the initiation and progression of substance-use disorders in these individuals.
